# Critical period of oxygen supplementation and invasive ventilation: implications for severe retinopathy of prematurity

**DOI:** 10.1186/s13052-024-01629-6

**Published:** 2024-04-01

**Authors:** Ho Jung Choi, Baek Sup Shin, Seung Han Shin, Ee-Kyung Kim, Han-Suk Kim

**Affiliations:** Department of Pediatrics, Seoul National University Children’s Hospital, Seoul, Republic of Korea

**Keywords:** Retinopathy of Prematurity, Preterm infants, Hyperoxia, Invasive ventilation

## Abstract

**Background:**

Several studies have identified graded oxygen saturation targets to prevent retinopathy of prematurity (ROP), a serious complication in preterm infants. We aimed to analyze the critical period of oxygen supplementation and/or invasive ventilation associated with severe ROP.

**Methods:**

This retrospective case-control study included neonates with a gestational age (GA) < 29 weeks. Participants were divided into two groups: treated retinopathy and untreated/no retinopathy. Time-weighted average FiO_2_ (TWAFiO_2_) and weekly invasive ventilation were compared between groups by postnatal age (PNA) and postmenstrual age (PMA). The association of treated retinopathy with TWAFiO_2_ and invasive ventilation was analyzed.

**Results:**

Data from 287 neonates were analyzed; 98 were treated for ROP and had lower GAs (25.5 vs. 27.4 weeks, *p* < 0.01) and lower birthweights (747.6 vs. 1014 g, *p* < 0.001) than those with untreated/no ROP. TWAFiO_2_ was higher from PMA 26–34 weeks, except for PMA 31 weeks in treated ROP, and higher in the first nine weeks of life in treated ROP. On multiple logistic regression, TWAFiO_2_ and invasive ventilation were associated with ROP treatment during the first seven weeks PNA. Invasive ventilation was associated with ROP treatment from PMA 26–31 weeks; no association was found for TWAFiO_2_ and PMA.

**Conclusions:**

Amount of oxygen supplementation and/or invasive ventilation during the first 7 weeks of life or up to 31 weeks PMA was associated with development of severe ROP. This period might be candidate timing for strict oxygen supplementation strategies in preterm infants, while concerns of mortality with low oxygen supplementation should be further explored.

**Supplementary Information:**

The online version contains supplementary material available at 10.1186/s13052-024-01629-6.

## Background

Retinopathy of prematurity (ROP) is a serious complication in preterm infants and can lead to blindness in severe cases. The incidence of ROP has been reported to be 60–70% in preterm infants with a birthweight of less than 1500 g or with extremely low gestational age (GA), with treatment required in one-fourth of cases [1–3]. The pathophysiology of the disease is abnormal growth of retinal blood vessels, which is associated with low GA and birthweight [4–6]. After preterm birth, excessive oxygenation via ventilatory support contributes to the development of ROP in the early stages by inhibiting retinal vascular development (phase I), followed by vasoproliferation induced by hypoxia in the avascular regions of the retina (phase II) [7, 8].

Low oxygen saturation targets have been associated with a reduction in the incidence of ROP, and several randomized controlled trials have been conducted to determine the optimal oxygen supplementation to prevent the development of ROP. The Surfactant, Positive Pressure, and Oxygenation Randomized Trial (SUPPORT) showed that the incidence of severe ROP was significantly lower in survivors with a lower oxygen saturation target of 85–89% [9], and a meta-analysis found reduced ROP treatment rates, but increased mortality at 36 weeks postmenstrual age (PMA) and at discharge [10]. Based on these results, the American Academy of Pediatrics (AAP) Committee on Fetuses and Newborns does not recommend a target oxygen saturation of less than 90% [11].

Several recent studies have investigated graded oxygen saturation targets based on PMA [10, 12, 13]. This strategy is based on the pathophysiology of ROP, as hyperoxia in phase I ROP and hypoxia in phase II ROP contribute to the development of ROP in preterm infants. However, studies investigating graded or biphasic oxygen-targeting strategies have been conducted based on the predetermined timing of oxygen saturation differences. So far, there is a lack of data on the duration of the critical period for development of severe ROP from hyperoxia in preterm infants based on the analysis of continuous data of respiratory support or oxygen supplementation. In this study, we aimed to analyze the critical period for oxygen supplementation and/or invasive ventilation with regard to the development of severe ROP in extremely preterm infants.

## Methods

This retrospective case-control study included neonates with a GA of < 29 weeks born between 2013 and 2021 who were admitted to the neonatal intensive care unit (NICU) of Seoul National University Children’s Hospital. Patients who died before discharge were excluded from the study. The participants were divided into two groups: (1) those with retinopathy with spontaneous regression (untreated ROP) or those without retinopathy, and (2) those who underwent surgery (treated ROP). ROP was diagnosed and staged according to the International Classification of Retinopathies of Prematurity [14]. Infants with a birthweight < 2,000 g or gestational age less than 32 weeks were initially screened for ROP at 4 weeks PNA or 31–32 weeks’ GA, whichever came first. The patients were treated for extraretinal fibrovascular proliferation above a certain stage 3, a positive finding, or aggressive ROP [14]. The study institution did not offer intravenous injections of bevacizumab, an anti-VEGF drug, during the study period. Data on GA, birthweight, sex, ROP stage, ROP treatment, length of invasive ventilation, length of hospitalization, and inspired oxygen concentrations were collected. The presence of other neonatal comorbid conditions including respiratory distress syndrome (RDS), bronchopulmonary dysplasia, persistent ductus arteriosus (PDA), sepsis, intraventricular hemorrhage, periventricular leukomalacia, necrotizing enterocolitis was also recorded. This study was approved by the Institutional Review Board of our institution (H-2210-131-1372). The requirement for informed consent was waived due to the retrospective study design.

Time-weighted average (TWAFiO_2_) was calculated as the area under the FiO_2_ versus the time plot on a weekly basis, and compared between groups according to PNA and PMA. TWAFiO_2_ during invasive oxygen supplementation, continuous nasal positive end-expiratory pressure, and high-flow nasal cannula (≥ 2 L/min) were included in the calculation. Each week, based on PNA and PMA, the categorical variable of invasive ventilation was determined if more than four days of oxygen supplementation via invasive ventilation was provided. The target SpO_2_ of the study population during oxygen supplementation was 90–95% regardless of PNA.

The chi-square test was used to compare categorical variables and the independent sample t-test was used to compare continuous variables. GA- and birthweight-adjusted p-values were calculated using adjusted proportion analysis or multiple linear regression analysis for demographic and clinical data. Multivariate logistic regression analysis was performed for ROP treatment on a weekly basis with respect to PMA and PNA, adjusted for GA, birthweight, and duration of invasive ventilation. Continuous variables are presented as mean ± standard deviation (SD). The results were considered statistically significant if the p-value was < 0.05.

## Results

Of the 287 infants analyzed, 98 were treated for ROP. Infants in the treated ROP group had a lower gestational age (25.5 vs. 27.4 weeks, *p* < 0.001) and lower birthweight (747.6 vs. 1014 g, *p* < 0.001) than those in the untreated/no ROP group (Table 1). After adjusting for GA and birthweight, the prevalence of RDS and treated PDA was significantly higher in the treated ROP group, and infants in the treated ROP group had significantly longer durations of invasive ventilation (46.3 vs. 13.1 days, *p* < 0.001) and hospitalization.

**Table 1:** Patient demographics and clinical characteristics

	No treated ROP (*n* = 189)	Treated ROP (*n* = 98)	p-value	*Adjusted p-value
GA (weeks)	27.4 (± 1.1)	25.5 (± 1.4)	*< 0.001*	*-*
Birthweight (g)	1014 (± 228.7)	747.6 (± 150.9)	*< 0.001*	*-*
SGA	10 (5.3%)	12 (12.2%)	*0.036*	*0.150*
Male	111 (58.7%)	47 (48.0%)	*0.082*	*0.260*
RDS	141 (74.6%)	95 (96.9%)	*< 0.001*	*0.013*
BPD	158 (83.6%)	95 (96.9%)	*0.001*	*0.400*
Treated PDA	102 (54.0%)	77 (78.6%)	*< 0.001*	*0.026*
Sepsis	22 (11.6%)	36 (36.7%)	*< 0.001*	*0.055*
IVH ≥ Gr 3	58 (30.7%)	47 (48.0%)	*0.004*	*0.332*
PVL	11 (5.8%)	5 (5.1%)	*0.801*	*0.721*
NEC	11 (5.8%)	10 (10.2%)	*0.176*	*0.801*
Invasive ventilation (days)	13.1 (± 18.8)	46.3 (± 45.1)	*< 0.001*	*< 0.001*
Hospital days	82.1 (± 23.1)	124.4 (± 54.5)	*< 0.001*	*< 0.001*

Values are presented as mean (± standard deviation) or number (%). *p-value of adjusted proportion adjusting GA and birthweight or correlation coefficient adjusting gestational age and birthweight. ROP, retinopathy of prematurity; GA, gestational age; SGA, small for gestational age; RDS, respiratory distress syndrome; BPD, bronchopulmonary dysplasia; PDA, patent ductus arteriosus; IVH, intraventricular hemorrhage; PVL, periventricular leukomalacia; NEC, necrotizing enterocolitis

In a weekly comparison based on PMA, TWAFiO_2_ was higher from PMA 26–34 weeks, except for PMA at 31 weeks, in the treated ROP group (Table 2). In the comparison based on PNA, TWAFiO_2_ was higher in the first nine weeks of life in the treated ROP group. In a subgroup analysis, the difference in TWAFiO_2_ was evident from PNA 2–7 weeks in infants born at 26–28 weeks GA, whereas differences were found at PMA 33 weeks (Supplementary Table 1, Additional file 1). In the subgroup of 23–25 weeks GA, differences were found at 26 weeks of PMA and at two, three, and five weeks of PNA.

**Table 2:** Time-weighted average FiO_2_ at each PMA and PNA

PMA	No treated ROP (*n* = 189)	Treated ROP (*n* = 98)	p-value	PNA	No treated ROP (*n* = 189)	Treated ROP (*n* = 98)	p-value
24 weeks	0.36 (0.21–0.73)	0.26 (0.24–0.34)	*0.517*	1st week	0.23 (0.22–0.26)	0.25 (0.22–0.29)	*0.003*
25 weeks	0.24 (0.22–0.28)	0.26 (0.22–0.36)	*0.301*	2nd week	0.23 (0.21–0.25)	0.27 (0.24–0.33)	*< 0.001*
26 weeks	0.24 (0.22–0.29)	0.28 (0.23–0.35)	*0.039*	3rd week	0.25 (0.21–0.27)	0.31 (0.27–0.38)	*< 0.001*
27 weeks	0.24 (0.22–0.29)	0.27 (0.24–0.32)	*0.004*	4th week	0.25 (0.22–0.29)	0.33 (0.27–0.38)	*< 0.001*
28 weeks	0.24 (0.22–0.3)	0.27 (0.22–0.34)	*0.047*	5th week	0.25 (0.21–0.3)	0.34 (0.28–0.39)	*< 0.001*
29 weeks	0.25 (0.22–0.3)	0.27 (0.23–0.34)	*0.031*	6th week	0.25 (0.21–0.3)	0.33 (0.27–0.37)	*< 0.001*
30 weeks	0.25 (0.22–0.31)	0.28 (0.23–0.36)	*0.014*	7th week	0.25 (0.21–0.3)	0.3 (0.26–0.36)	*0.002*
31 weeks	0.25 (0.22–0.31)	0.26 (0.22–0.34)	*0.160*	8th week	0.26 (0.23–0.3)	0.29 (0.24–0.34)	*0.012*
32 weeks	0.25 (0.21–0.32)	0.29 (0.23–0.38)	*0.017*	9th week	0.26 (0.22–0.3)	0.3 (0.24–0.35)	*0.014*
33 weeks	0.25 (0.21–0.33)	0.28 (0.24–0.37)	*0.004*	10th week	0.26 (0.22–0.32)	0.29 (0.25–0.32)	*0.069*
34 weeks	0.25 (0.21–0.31)	0.27 (0.24–0.35)	*0.010*	11th week	0.28 (0.25–0.31)	0.28 (0.25–0.32)	*0.583*
35 weeks	0.26 (0.21–0.32)	0.27 (0.24–0.33)	*0.223*	12th week	0.3 (0.25–0.32)	0.29 (0.25–0.32)	*0.944*

Values are presented as median (interquartile range). FiO_2_, Fraction of inspired oxygen; PMA, postmenstrual age; PNA, postnatal age; ROP, retinopathy of prematurity

**Fig. 1:**
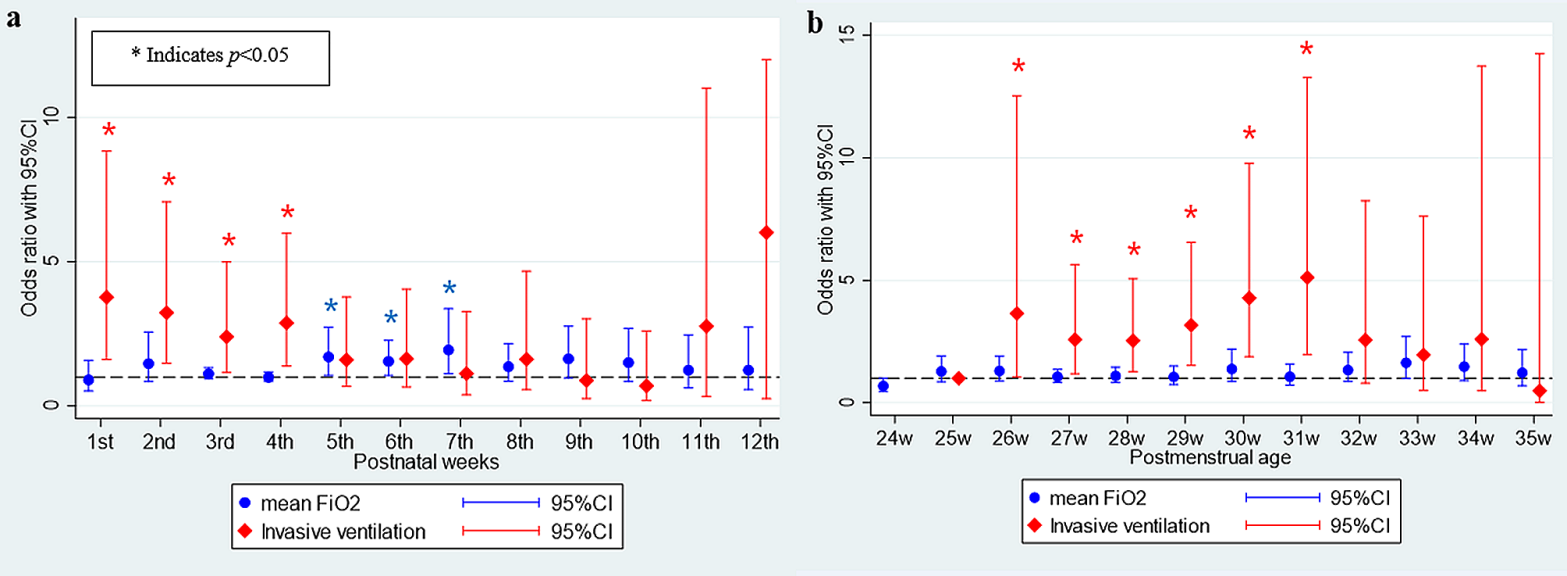
Association of time-weighted average FiO_2_ and invasive ventilation with treated ROP. (**a**) at each postnatal age and (**b**) at each postmenstrual age. Adjusted for gestational age, birthweight and invasive ventilation at each week (*p < 0.05). FiO_2,_ Fraction of inspired oxygen; ROP, retinopathy of prematurity

In multivariate logistic regression analysis adjusted for GA, birthweight, and weekly invasive ventilation, invasive ventilation was associated with treated ROP from 1 to 4 weeks PNA, and higher supplemental oxygen (as an increase in FiO_2_ by 0.1) was associated with treated ROP from 5 to 7 weeks PNA (Fig. 1a). Treated ROP was associated with invasive ventilation from 26 to 31 weeks PMA, whereas the level of supplemental oxygen was not associated with the treated ROP (Fig. 1b).

## Discussion

This study investigated the critical postnatal periods for the development of severe ROP influenced by oxygen supplementation or invasive ventilation in the setting of a target saturation of 90–95% using an institutional protocol. TWAFiO_2_ or invasive ventilation was associated with ROP treatment in the first 7 weeks PNA. In terms of PMA, invasive ventilation was associated with ROP from PMA 26–31 weeks, whereas no association between TWAFiO_2_ and treated ROP was found.

One of the notable findings elucidated in the present study pertains to the association between invasive ventilation and severe ROP even after adjusting for the level of oxygen given to the patients. While the existing literature has underscored the link between mechanical ventilation and ROP, prior investigations have not scrutinized the independent impact of invasive ventilation, distinct from the quantity of oxygen concentration delivered [15–17]. Although non-invasive ventilation can effectively support patient’s respiratory support, there are disadvantages of oxygenation supplementation compared with invasive ventilation in several aspects [18]. Patient-dependent factors, such as minute ventilation, amount of mouth breathing, leakage around the patient-device interface, and the relative duration of inspiration and expiration, can influence the fractional inspired oxygen concentration, thereby compromising the intended oxygen delivery efficacy [19, 20].

As the present study investigated the level of oxygen given to the patients with regard to the development of severe ROP, considering the mode of ventilation that affects effective oxygen delivery was crucial in this study. Our findings were also supported by the results of an *ad hoc* analysis, comparing the incidence of severe ROP between patients receiving invasive ventilation and non-invasive ventilation in the subgroup stratified by weekly TWAFiO_2_ (< 0.3 and ≥ 0.3). Across both subgroup delineations, there was a higher incidence of severe ROP in the group receiving invasive ventilation early in life (Supplementary Table 2, Additional file 1).

After the AAP stated that a lower target saturation strategy for the prevention of ROP could not account for the higher incidence of death in preterm infants, several studies have attempted to demonstrate the effect of a graded saturation target strategy in the prevention of ROP without increasing mortality in preterm infants [10, 12, 13]. The hypothesis that the target saturation varies with age is based on the pathophysiology of ROP, which comprises two phases. In phase I, early hyperoxia induces attenuation of retinal vascular growth and vaso-obliteration. This is followed by the hypoxia-induced phase II vasoproliferation in the avascular areas of the retina [7, 8]. However, these studies evaluated specific periods of oxygen restriction based on the PMA rather than on the PNA. A retrospective study in 2016 applied a graded SpO_2_ target based on PMA (83–89% until 32^6/7^ weeks, 90–94% until 35^6/7^ weeks, and > 94% at ≥ 36 weeks PMA) and showed decreased rates of severe ROP and laser surgery without increasing mortality [12]. Another study by Shukla et al. compared a graded SpO_2_ target (85–92% until 33^6/7^ weeks PMA and 95% at ≥ 34 weeks PMA) with a constant target (91–95%) [13]. The results of the present study also showed that setting the period before and after PMA to 32–33 weeks might be rational, as severe ROP was associated with higher level of oxygen supplementation or invasive ventilation before 32 weeks PMA. Moreover, our study investigated the period of interest in terms of PNA. PNA-based evaluation could provide additional information, as preterm infants of various GAs were included in the study. As the oxygen concentration in the atmosphere is higher than that in the intrauterine environment, it can be assumed that virtually all preterm infants are exposed to oxygen stress from the moment they are born.

In recent years, there has been an increasing focus in preclinical and clinical research on target therapies aimed at decoupling oxygenation and vascularization [21, 22]. Given the variability in VEGF levels between phase I and phase II of ROP pathogenesis, it is valuable to identify specific target periods where therapeutic interventions can maximize their efficacy in decoupling the effects of oxygen on vascular development. Hence, the results of the current study may provide insights into determining the optimal timing of these therapeutic agents in future applications.

The first limitation of the present study is that different oxygen saturations were not compared. Instead, we sought to evaluate the effect of the actual amount of oxygen supplementation and invasive ventilation on severe ROP. The analysis of these factors is a different approach from previous studies that set different oxygen saturation targets [10, 12, 13]. However, SpO_2_ can be affected by other systemic conditions such as pulmonary hypertension, blood pressure, or lung disease. In fact, the treated ROP group in this study tended to have lower mean SpO_2_ values, even though they were supplemented with higher oxygen levels (Supplementary Table 3, Additional file 1). Moreover, because oxygen can be delivered both invasively and noninvasively, and the level of oxygen can be determined accordingly, the association of invasive ventilation at a specific PNA or PMA with the development of severe ROP was also considered. Considering the graded oxygen saturation target strategy, the first seven weeks of life or up to PMA 31 weeks could be a candidate period for a lower saturation target. As infants who died before discharge were excluded from the study population, the concerns regarding mortality in the lower saturation target group, as seen in previous studies such as the SUPPORT trial, could not be addressed in this study. The primary objective of the present study was to investigate the vulnerable period in the development of severe ROP in preterm infants.

Another limitation of this study is that we did not calculate the actual amount of oxygen delivered during non-invasive ventilation. However, the aim of this study was not to determine the exact amount of oxygenation that increases the risk of severe ROP but rather to investigate the period of significance affected by oxygen supplementation and invasive ventilation. Therefore, we adjusted for invasive ventilation in each period.

## Conclusion

Differences in required oxygen supplementation and/or invasive ventilation between the untreated and treated ROP groups were present in our population sample. The first 7 weeks of life or up to PMA 31 weeks may be a candidate period for a lower saturation target when considering graded or biphasic oxygen saturation target strategies for reducing ROP. Further prospective studies are required to determine the critical period for oxygen restriction policies for neonates born before 29 weeks GA.

### Electronic supplementary material

Below is the link to the electronic supplementary material.

Supplementary Material 1

Supplementary Material 2

## Data Availability

The datasets analyzed during the current study are available from the corresponding author on reasonable request.

## Declarations

The requirement for informed consent was waived due to the retrospective study design.

## Abbreviations

AAP: American Academy of Pediatrics
BPD: Bronchopulmonary dysplasia
GA: Gestational age
IVH: Intraventricular hemorrhage
NEC: Necrotizing enterocolitis
NICU: Neonatal intensive care unit
PDA: Patent ductus arteriosus
PMA: Postmenstrual age
PNA: Postnatal age
PVL: Periventricular leukomalacia
RDS: Respiratory distress syndrome
ROP: Retinopathy of prematurity
SGA: Small for gestational age
SUPPORT: Surfactant, Positive Pressure, and Oxygenation Randomized Trial
TWAFiO_2_: Time-weighted average FiO2
