# Supplementary material for: Critical period of oxygen supplementation and invasive ventilation: implications for severe retinopathy of prematurity
Source: Ital J Pediatr. 2024 Apr 1;50:58. doi: 10.1186/s13052-024-01629-6 (PMC10985977; doi:10.1186/s13052-024-01629-6)
Supplement: Supplementary file 1 — Supplementary Material 1 [file 13052_2024_1629_MOESM1_ESM.docx]

**Additional file 1**

**Critical Period of Oxygen Supplementation and Invasive Ventilation: Implications for Severe Retinopathy of Prematurity**

Italian Journal of Pediatrics

Ho Jung Choi^1^, Baek Sup Shin^1^, Seung Han Shin^1^*, Ee-Kyung Kim^1^ & Han-Suk Kim^1^

^1^ Department of Pediatrics, Seoul National University College of Medicine, Seoul, Republic of Korea

* Correspondence:

Seung Han Shin, MD, PhD

revival421@snu.ac.kr

Supplementary Table 1. Time-weighted average FiO_2_ at each PMA and PNA in the subgroup of gestational age

| PMA | No treated ROP (n=189) | Treated ROP  (n=98) | p value | PNA | No treated ROP (n=189) | Treated ROP  (n=98) | p value |
| --- | --- | --- | --- | --- | --- | --- | --- |
| **Subgroup: GA 23~25 weeks (n=82)** | | | | | | | |
|  | n=21 | n=61 |  |  | n=21 | n=61 |  |
| 24 weeks | 0.36 (0.21-0.73) | 0.26 (0.24-0.34) | *0.517* | 1^st^ week | 0.24 (0.22-0.31) | 0.26 (0.22-0.29) | *0.778* |
| 25 weeks | 0.24 (0.22-0.28) | 0.26 (0.22-0.36) | *0.301* | 2^nd^ week | 0.25 (0.23-0.27) | 0.3 (0.26-0.34) | *0.003* |
| 26 weeks | 0.23 (0.22-0.29) | 0.28 (0.24-0.35) | *0.017* | 3^rd^ week | 0.29 (0.26-0.32) | 0.32 (0.28-0.39) | *0.013* |
| 27 weeks | 0.27 (0.23-0.29) | 0.28 (0.26-0.36) | *0.156* | 4^th^ week | 0.3 (0.26-0.37) | 0.34 (0.29-0.39) | *0.116* |
| 28 weeks | 0.28 (0.27-0.35) | 0.29 (0.24-0.37) | *0.895* | 5^th^ week | 0.3 (0.26-0.34) | 0.36 (0.3-0.41) | *0.023* |
| 29 weeks | 0.27 (0.25-0.35) | 0.3 (0.25-0.35) | *0.902* | 6^th^ week | 0.3 (0.26-0.36) | 0.34 (0.29-0.4) | *0.112* |
| 30 weeks | 0.31 (0.23-0.32) | 0.29 (0.24-0.38) | *0.521* | 7^th^ week | 0.29 (0.25-0.34) | 0.32 (0.28-0.38) | *0.092* |
| 31 weeks | 0.3 (0.25-0.36) | 0.3 (0.24-0.38) | *0.904* | 8^th^ week | 0.29 (0.24-0.33) | 0.31 (0.26-0.36) | *0.238* |
| 32 weeks | 0.27 (0.24-0.33) | 0.32 (0.27-0.42) | *0.101* | 9^th^ week | 0.27 (0.21-0.34) | 0.31 (0.25-0.37) | *0.098* |
| 33 weeks | 0.27 (0.23-0.33) | 0.29 (0.25-0.37) | *0.428* | 10^th^ week | 0.25 (0.21-0.32) | 0.29 (0.25-0.33) | *0.109* |
| 34 weeks | 0.25 (0.21-0.32) | 0.28 (0.25-0.38) | *0.117* | 11^th^ week | 0.28 (0.25-0.33) | 0.28 (0.25-0.32) | *0.667* |
| 35 weeks | 0.31 (0.21-0.32) | 0.27 (0.24-0.34) | *0.656* | 12^th^ week | 0.32 (0.3-0.33) | 0.28 (0.24-0.32) | *0.304* |
| **Subgroup: GA 26~28 weeks (n=205)** | | | | | | | |
|  | n=168 | n=37 |  |  | n=168 | n=37 |  |
| 26 weeks | 0.25 (0.23-0.3) | 0.25 (0.21-0.31) | *0.773* | 1^st^ week | 0.23 (0.22-0.25) | 0.24 (0.22-0.27) | *0.207* |
| 27 weeks | 0.23 (0.22-0.29) | 0.25 (0.22-0.29) | *0.313* | 2^nd^ week | 0.23 (0.21-0.25) | 0.25 (0.23-0.29) | *<0.001* |
| 28 weeks | 0.24 (0.22-0.28) | 0.25 (0.22-0.27) | *1.000* | 3^rd^ week | 0.25 (0.21-0.27) | 0.28 (0.25-0.33) | *<0.001* |
| 29 weeks | 0.25 (0.22-0.29) | 0.25 (0.22-0.31) | *0.482* | 4^th^ week | 0.25 (0.21-0.28) | 0.31 (0.24-0.36) | *<0.001* |
| 30 weeks | 0.25 (0.22-0.3) | 0.26 (0.23-0.31) | *0.178* | 5^th^ week | 0.24 (0.21-0.28) | 0.29 (0.24-0.35) | *<0.001* |
| 31 weeks | 0.25 (0.21-0.3) | 0.25 (0.21-0.33) | *0.929* | 6^th^ week | 0.23 (0.21-0.28) | 0.29 (0.22-0.35) | *<0.001* |
| 32 weeks | 0.25 (0.21-0.31) | 0.27 (0.21-0.35) | *0.662* | 7^th^ week | 0.25 (0.21-0.28) | 0.28 (0.23-0.34) | *0.009* |
| 33 weeks | 0.25 (0.21-0.32) | 0.27 (0.23-0.37) | *0.038* | 8^th^ week | 0.25 (0.22-0.3) | 0.24 (0.21-0.31) | *0.962* |
| 34 weeks | 0.25 (0.21-0.31) | 0.26 (0.24-0.34) | *0.116* | 9^th^ week | 0.26 (0.22-0.3) | 0.28 (0.21-0.3) | *0.518* |
| 35 weeks | 0.26 (0.21-0.33) | 0.27 (0.23-0.33) | *0.410* | 10^th^ week | 0.26 (0.22-0.32) | 0.27 (0.24-0.3) | *0.687* |

Values are presented as median (interquartile range). FiO_2_, Fraction of inspired oxygen; PMA, postmenstrual age; PNA, postnatal age; ROP, retinopathy of prematurity; GA, gestational age.

Supplementary Table 2. Incidence of treated ROP according to the mode of ventilation at each PMA and PNA in each subgroup based on TWAFiO_2_

| PMA | NIV | IV | p-value | PNA | NIV | IV | p-value | |
| --- | --- | --- | --- | --- | --- | --- | --- | --- |
| **Subgroup: TWAFiO_2_ <0.3** | | | | | | | |  |
| 26 weeks | 23.5% | 62.9% | *0.006* | 1^st^ week | 12.9% | 60.8% | *0.000* | |
| 27 weeks | 29.4% | 50.6% | *0.028* | 2^nd^ week | 13.9% | 54.4% | *1.000* | |
| 28 weeks | 21.4% | 43.6% | *0.002* | 3^rd^ week | 16.8% | 52% | *0.000* | |
| 29 weeks | 19.4% | 50.9% | *0.000* | 4^th^ week | 18.2% | 48% | *0.003* | |
| 30 weeks | 24.6% | 60.6% | *0.000* | 5^th^ week | 21% | 66.7% | *0.002* | |
| 31 weeks | 26.1% | 61.1% | *0.005* | 6^th^ week | 25% | 61.5% | *0.009* | |
| 32 weeks | 28% | 62.5% | *0.055* | 7^th^ week | 33.6% | 75% | *0.009* | |
| 33 weeks | 38% | 57.1% | *0.423* | 8^th^ week | 44.7% | 71.4% | *0.085* | |
| 34 weeks | 34.1% | 66.7% | *0.282* | 9^th^ week | 52.3% | 63.6% | *0.533* | |
| 35 weeks | 39.1% | 0% | *1.000* | 10^th^ week | 66.2% | 50% | *0.444* | |
| **Subgroup: TWAFiO_2_ ≥0.3** | | | | | | | |  |
| 26 weeks | 25% | 74.3% | *0.078* | 1^st^ week | 0% | 45.7% | *0.250* | |
| 27 weeks | 20% | 63.9% | *0.028* | 2^nd^ week | 0% | 80.4% | *0.003* | |
| 28 weeks | 28.1% | 55.3% | *0.030* | 3^rd^ week | 27.3% | 77.6% | *0.002* | |
| 29 weeks | 34.2% | 65.5% | *0.014* | 4^th^ week | 43.3% | 79.1% | *0.001* | |
| 30 weeks | 32.1% | 70.6% | *0.010* | 5^th^ week | 47.1% | 76.6% | *0.006* | |
| 31 weeks | 30.6% | 81.3% | *0.001* | 6^th^ week | 55.1% | 81% | *0.013* | |
| 32 weeks | 32.7% | 66.7% | *0.048* | 7^th^ week | 65.9% | 78.8% | *0.301* | |
| 33 weeks | 39.7% | 64.3% | *0.092* | 8^th^ week | 67.7% | 77.8% | *0.408* | |
| 34 weeks | 38.1% | 60% | *0.381* | 9^th^ week | 75% | 78.6% | *1.000* | |
| 35 weeks | 37.5% | 50% | *1.000* | 10^th^ week | 75% | 84.6% | *0.698* | |

Values are presented as median (interquartile range). ROP, retinopathy of prematurity; TWAFiO_2_, time-weighted average FiO_2_; PMA, postmenstrual age; PNA, postnatal age; NIV, non-invasive ventilation; IV, invasive ventilation

Supplementary Table 3. Oxygen saturation (%) at each PMA and PNA in the study group

| PMA | No treated ROP (n=189) | Treated ROP (n=98) | p value | PNA | No treated ROP (n=189) | Treated ROP (n=98) | p value |
| --- | --- | --- | --- | --- | --- | --- | --- |
| 24 weeks | 92.2 (76.2-96.9) | 94.3 (92.9-96.1) | *0.333* | 1^st^ week | 96.1 (94.5-97) | 94.9 (93.4-96.2) | *<0.001* |
| 25 weeks | 95.3 (94.8-96.7) | 93.9 (92.1-95.8) | *0.048* | 2^nd^ week | 94.9 (93.6-96.7) | 93.5 (91.9-95) | *<0.001* |
| 26 weeks | 94.4 (92.2-95.9) | 94.5 (92.4-95.9) | *0.625* | 3^rd^ week | 94.6 (93.1-96.4) | 93 (91.2-94.4) | *<0.001* |
| 27 weeks | 94.8 (92.1-96.2) | 94.2 (92.1-95.3) | *0.257* | 4^th^ week | 95.2 (93-97) | 92.9 (90.6-94.4) | *<0.001* |
| 28 weeks | 94.8 (92.5-96.8) | 93.9 (91.7-96.3) | *0.051* | 5^th^ week | 95.6 (93.5-97.3) | 92.6 (90.7-94.3) | *<0.001* |
| 29 weeks | 94.2 (92.6-96.7) | 94 (91.7-96.7) | *0.331* | 6^th^ week | 96.1 (94-97.8) | 93.1 (90.9-95) | *<0.001* |
| 30 weeks | 94.4 (92.7-96.2) | 94.2 (92.1-96.5) | *0.384* | 7^th^ week | 96.7 (94.3-97.7) | 93.4 (90.8-95.5) | *<0.001* |
| 31 weeks | 95 (92.8-97) | 94.7 (92.2-97) | *0.528* | 8^th^ week | 96.6 (94.8-97.7) | 94 (91.9-96.1) | *<0.001* |
| 32 weeks | 95.3 (93.3-97.4) | 95.2 (92.9-97.2) | *0.782* | 9^th^ week | 96.8 (95.1-97.7) | 94.6 (92.6-96.6) | *<0.001* |
| 33 weeks | 95.8 (94-97.5) | 95.1 (93.5-97.3) | *0.234* | 10^th^ week | 96.4 (95-97.7) | 94.9 (93.6-96.5) | *<0.001* |
| 34 weeks | 96.2 (93.9-97.6) | 95.6 (93.5-97.5) | *0.312* | 11^th^ week | 96.5 (94.9-97.7) | 95.1 (93.5-96.8) | *<0.001* |
| 35 weeks | 96.3 (94.3-97.6) | 95.8 (93.6-97.5) | *0.309* | 12^th^ week | 96.6 (95.4-97.7) | 95.1 (93.6-96.6) | *<0.001* |

Values are presented as median (interquartile range). PMA, postmenstrual age; PNA, postnatal age; ROP, retinopathy of prematurity.
