# Supplementary material for: Critical period of oxygen supplementation and invasive ventilation: implications for severe retinopathy of prematurity
Source: Ital J Pediatr. 2024 Apr 1;50:58. doi: 10.1186/s13052-024-01629-6 (PMC10985977; doi:10.1186/s13052-024-01629-6)
Supplement: Supplementary file 2 — Supplementary Material 2 [file 13052_2024_1629_MOESM2_ESM.docx]

[2024.02.17]

Professor Giovanni Corsello, PhD, MD

Editor-in-Chief

*Italian Journal of Pediatrics*

Dear Dr Corsello:

We wish to re-submit the manuscript titled “Critical Period of Oxygen Supplementation and Invasive Ventilation: Implications for Severe Retinopathy of Prematurity.” The manuscript ID is ITJP-D-23-00720R1.

We thank you and the reviewers for your thoughtful suggestions and insights. The manuscript has benefited from these insightful suggestions. I look forward to working with you and the reviewers to move this manuscript closer to publication in the *Italian Journal of Pediatrics*.

The manuscript has been rechecked and the necessary changes have been made in accordance with the reviewers’ suggestions. The changes to the manuscript are indicated by red-colored text. The responses to all comments have been prepared and attached herewith.

Thank you for your consideration. I look forward to hearing from you.

Sincerely,

Seung Han Shin, MD, PhD

Department of Pediatrics, Seoul National University Children’s Hospital, Seoul, Republic of Korea

Email: revival421@snu.ac.kr
